# Supplementary figures and images for: Inhibition of histone H3-H4 chaperone pathways rescues C. elegans sterility by H2B loss
Source: PLoS Genet. 2022 Jun 9;18(6):e1010223. doi: 10.1371/journal.pgen.1010223 (PMC9216614; doi:10.1371/journal.pgen.1010223)

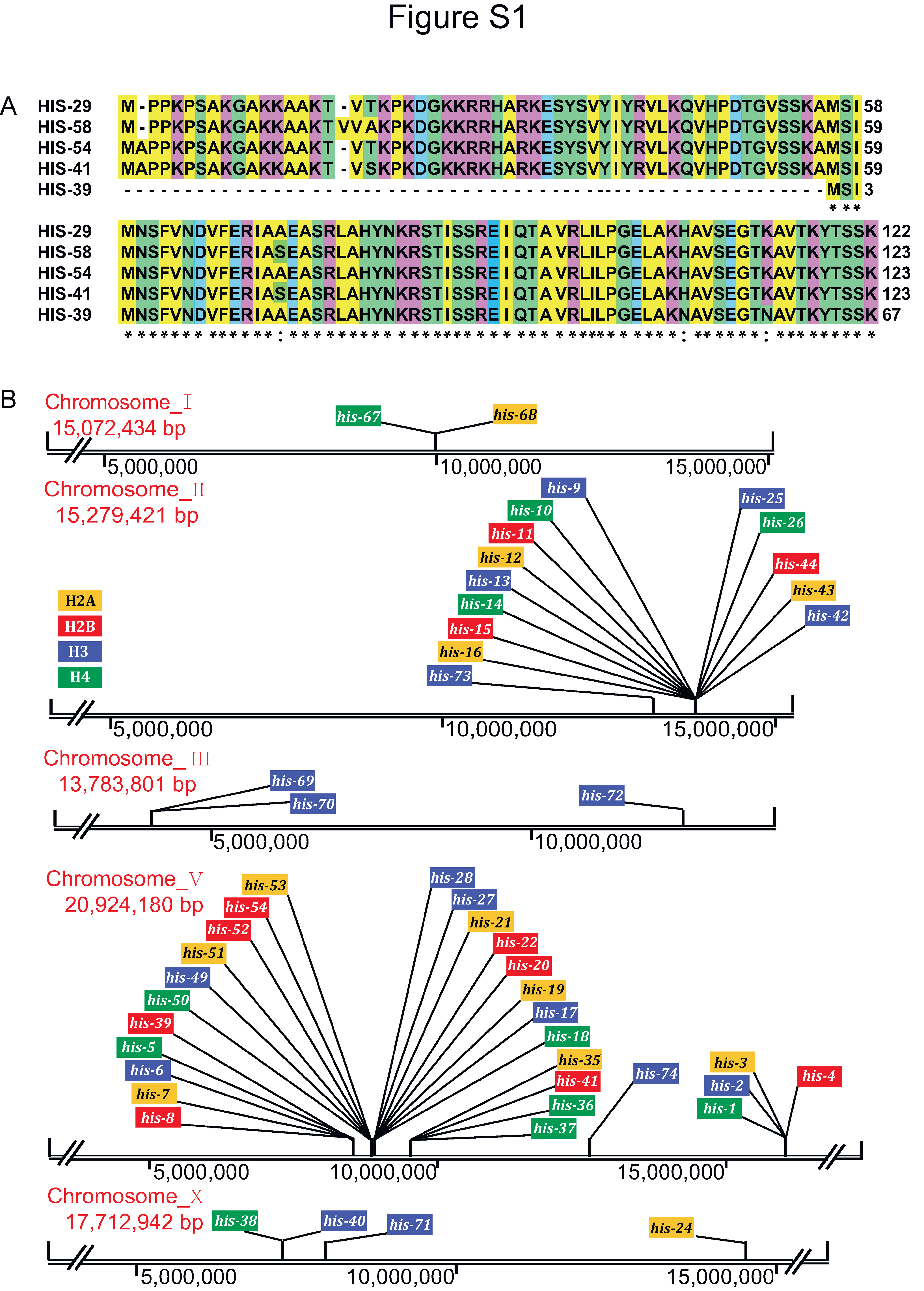

Supplement: S1 Fig — Related to Fig 1. (A) Multiple protein sequence alignment of five C. elegans (Ce) H2B proteins. An asterisk (*) indicates positions 100% conserved in the alignment, a colon (:) indicates conservation between groups of strongly similar properties. (B) Genomic loci of all histone genes in C. elegans. Yellow boxes indicate H2A, red boxes indicate H2B, blue boxes indicate H3, and green boxes indicate H4. (TIF) [file pgen.1010223.s001.tif]

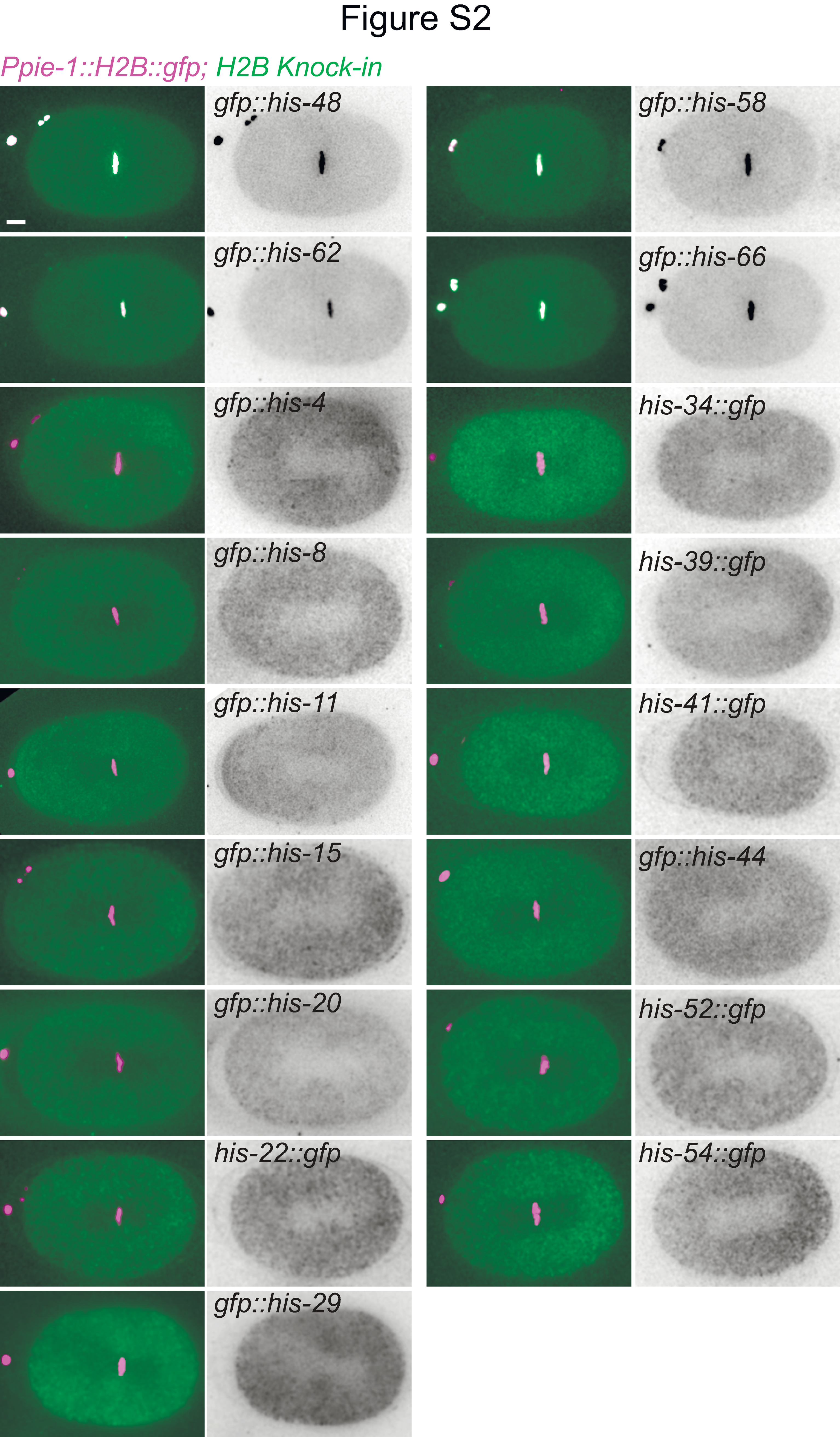

Supplement: S2 Fig — Related to Fig 1. The HIS-58 subfamily H2B expresses in one-cell stage embryos. Embryo fluorescence was visualized by GFP::H2B (green) and Ppie-1::H2B::mCherry (magenta). Scale bar, 5 μm. (TIF) [file pgen.1010223.s002.tif]

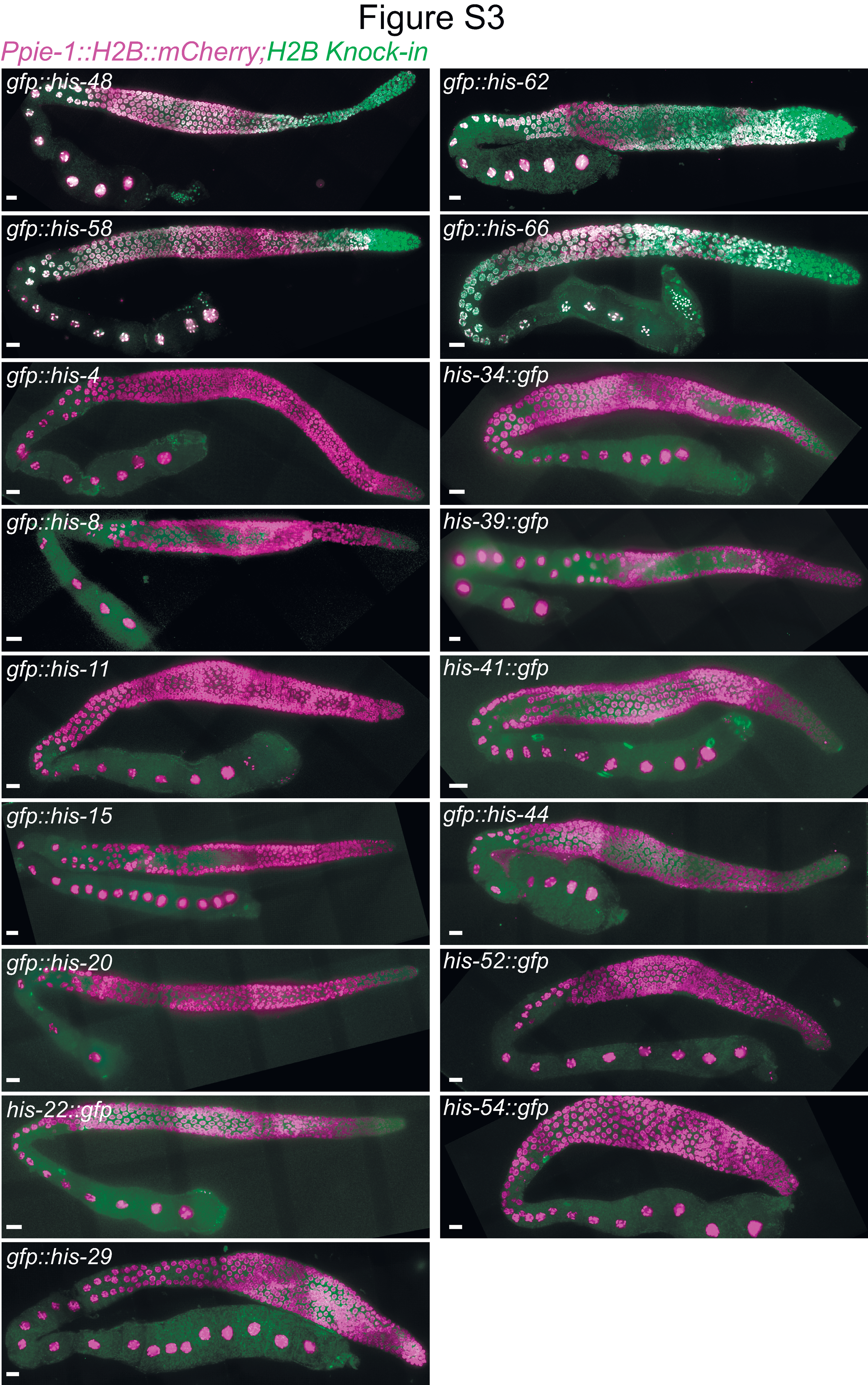

Supplement: S3 Fig — Related to Fig 1. The HIS-58 subfamily H2B expresses in C. elegans germlines. Germline fluorescence was visualized by GFP::H2B (green) and Ppie-1::H2B::mCherry (magenta). Scale bar, 10 μm. (TIF) [file pgen.1010223.s003.tif]

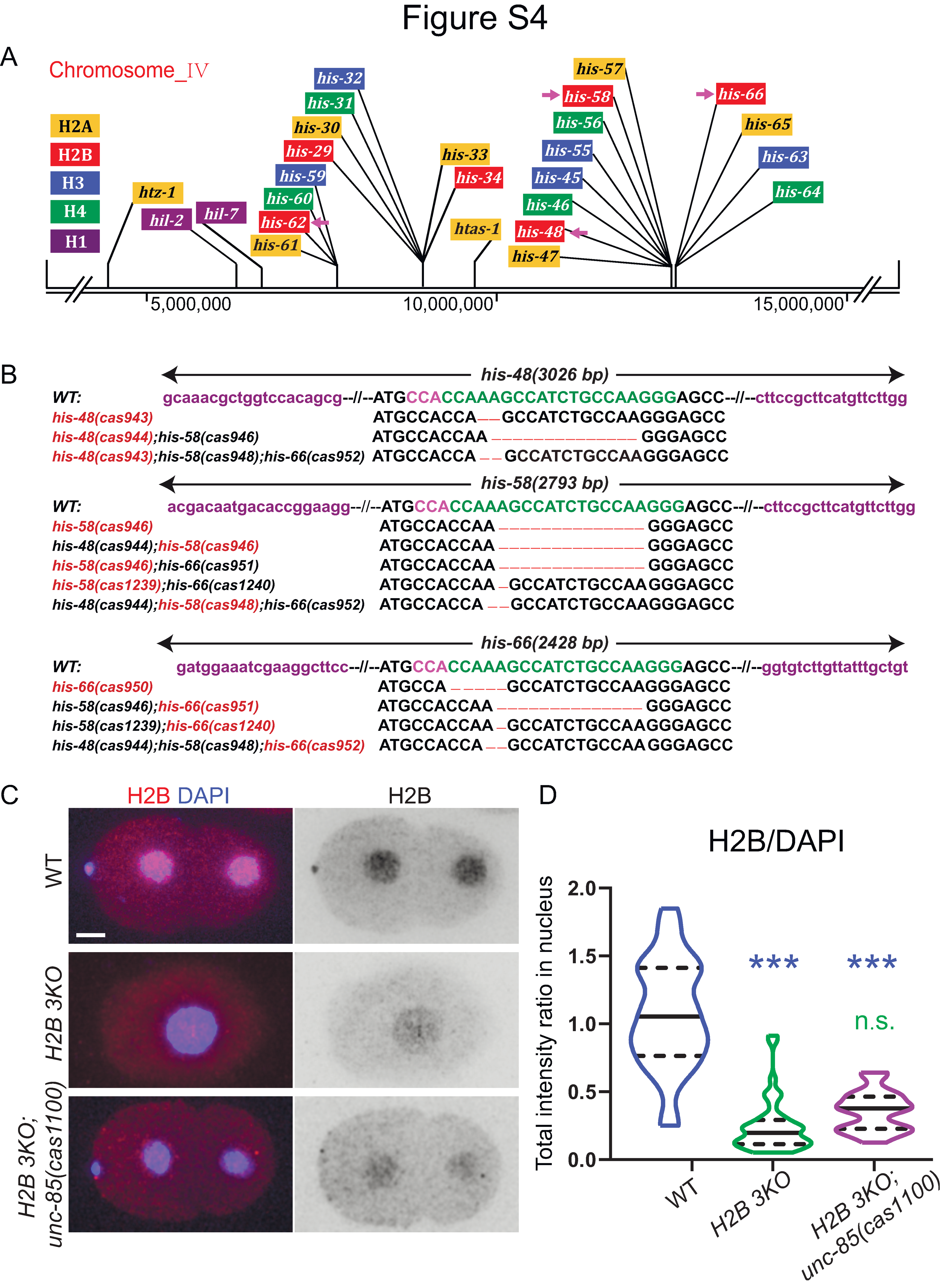

Supplement: S4 Fig — Related to Fig 1. (A) Histone gene clusters on chromosome IV. Magenta arrows indicate four germline H2B genes. (B) DNA sequences of germline H2B mutants generated via CRISPR-Cas9. The 20 nt target sequences and the following PAMs (NGG) are highlighted in green and magenta, separately. Purple sequence, PCR primers; Red dashed lines, deleted nucleotides. (C) Immunofluorescence images with the anti-H2B antibody of WT, H2B 3KO, and H2B 3KO; unc-85(cas1100) quadruple mutant embryos. DAPI stained nuclei. Scale bar, 5 μm. (D) Quantification of histone H2B fluorescence intensity ratio relative to DAPI of WT, H2B 3KO, and H2B 3KO; unc-85(cas1100) embryos. N = 28–43. Data are presented as mean ± SD. Statistical significance compared with the control with a matching color code is based on Student’s t-test, n.s., not significant, ***p < 0.001. (TIF) [file pgen.1010223.s004.tif]

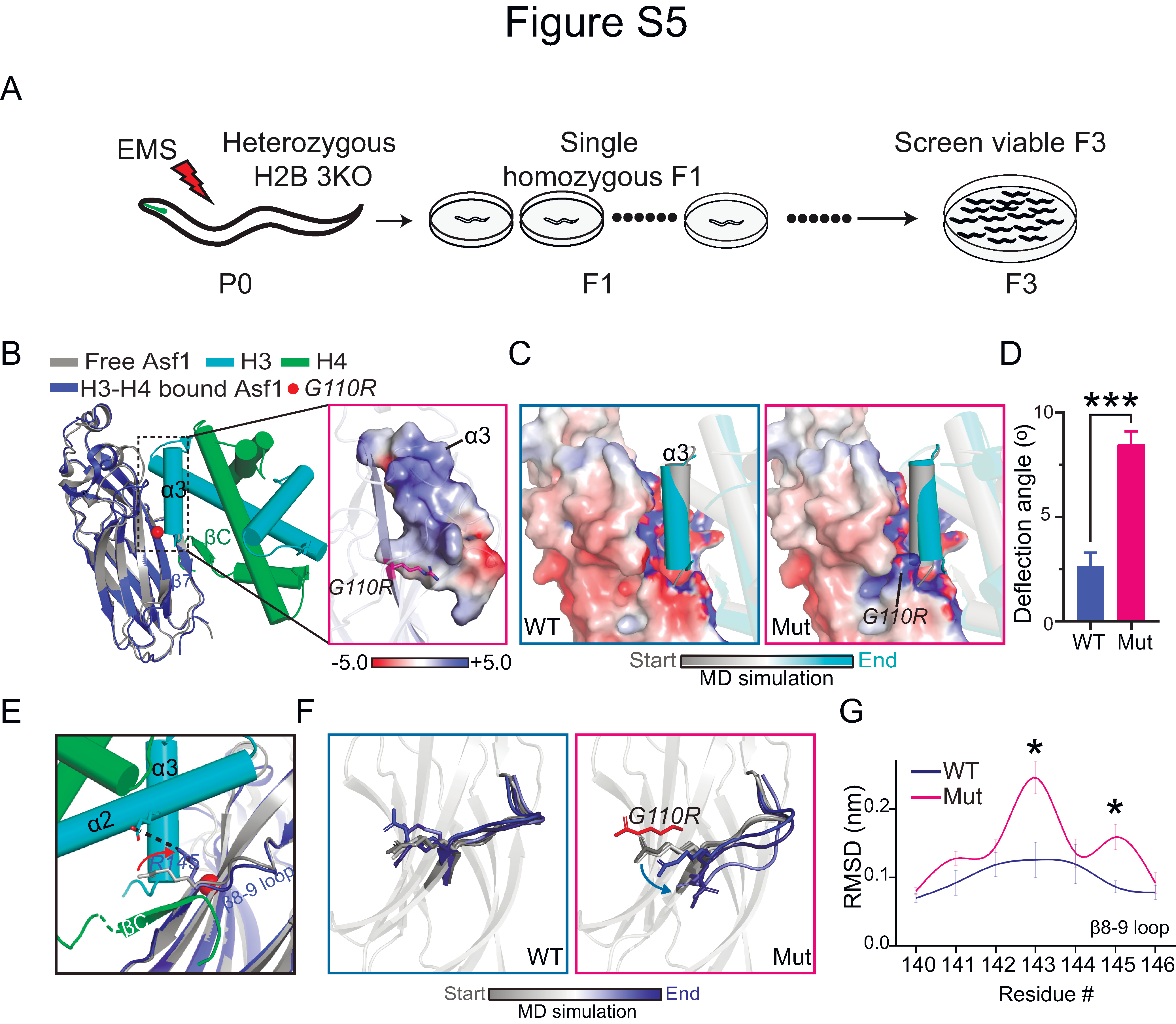

Supplement: S5 Fig — Related to Figs 1 and 2. (A) A flowchart of the suppressor screening. H2B 3KO animals are sterile (Ste). At the late L4 stage, animals were mutagenized with ethyl methanesulfonate (EMS). Viable F3 animals were candidate suppressors. The mutant genes were cloned using single-nucleotide polymorphism mapping combined with whole-genome sequencing. (B) Superposition of free and histone-bound Asf1 structures with the Asf1-H3 binding interface highlighted. The mutation site (G110R) within the H3-binding cleft of Asf1 is situated closely to the positively charged H3 surface in the Asf1-H3-H4 complex structure. (C) Molecular dynamic (MD) simulation of Asf1-H3-H4 complex suggests the G110R-induced dissociation of H3-H4 from Asf1. G110R introduced a positively charged surface bulge within the H3-docking pocket, and as a result, the α3 helix of H3 responsible for Asf1 binding underwent a significant deflection away from Asf1 shown in the time-lapse images of the simulation of mutant structure. (D) Statistical analysis of the deflection angles of the α3 helix in the MD analysis. (E) Structural representation of the Asf1-H4 interface showing the rotation of Asf1 R145 required for H4 binding. (F) Asf1 G110R structure shows an increase of movement within the β8–9 loop, including R145, which appears to hinder the H4 binding. Time-lapse images highlight the changes in the β8–9 loop throughout the simulation. (G) Statistical analysis of the structure deviation of residues in the β8–9 loop in the simulation. Values are representative of three independent simulations, and error bars indicate mean ± SD, *p<0.05, ***p<0.001. Asf1-H3/H4 complex (PDB code: 2HUE) and free Asf1 (PDB: 1ROC) structures were used for MD and structural analysis. (TIF) [file pgen.1010223.s005.tif]

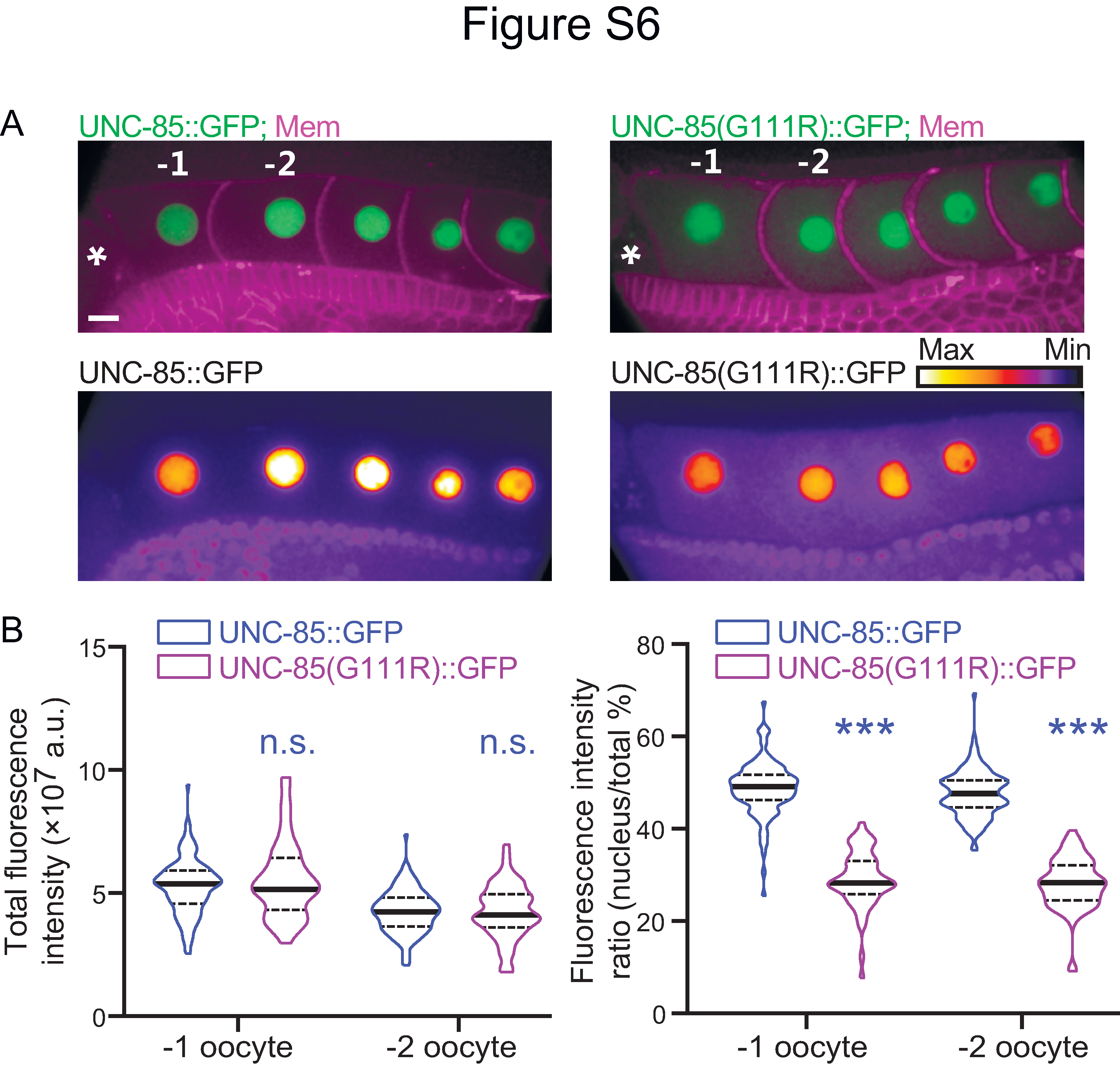

Supplement: S6 Fig — Related to Fig 2. (A) Fluorescence time-lapse images (up) and heatmaps (down) of UNC-85::GFP (left) and UNC-85::GFPG111R (right) in the germline at the day one adult stage. GFP images are shown as heatmaps below the merged images. Scale bar, 5 μm. (B) Quantifications of GFP-tagged UNC-85 total fluorescence intensity in -1 and -2 oocyte (left) and intensity ratio of the nucleus to the cytoplasm (right). Data are presented as mean ± SD (error bars). Statistical significance based on two-way ANOVA, n.s., not significant, ***p < 0.001. N = 57–117. (TIF) [file pgen.1010223.s006.tif]

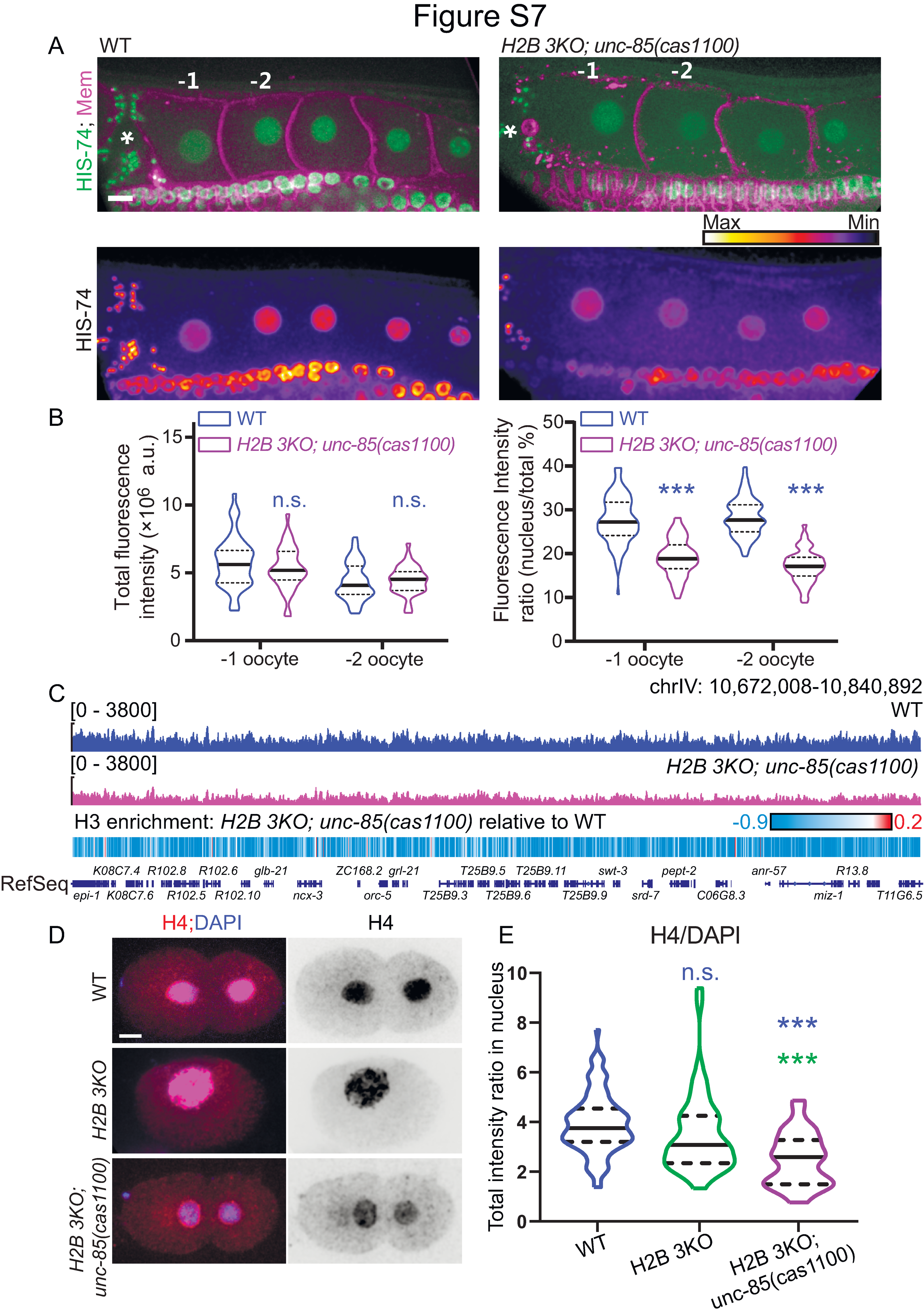

Supplement: S7 Fig — Related to Fig 4. (A) Fluorescence time-lapse images of GFP-tagged HIS-74 with mCherry-tagged (magenta) membrane of the germline in WT (left) or H2B 3KO; unc-85(cas1100) quadruple mutant (right) at the day-one adult stage. GFP images are shown as heatmaps below merged images. Scale bar, 5 μm. (B) Quantifications of GFP-tagged HIS-74 total fluorescence intensity in -1 and -2 oocyte (left) and intensity ratio of the nucleus to the cytoplasm (right). Data are presented as mean ± SD (error bars), statistical significance based on two-way ANOVA, n.s., not significant, ***p < 0.001. N = 37–52. (C) Genome tracks show H3 signals of a representative genomic locus (Chr IV: 10,627,008–10,840,892) in WT (up) and H2B 3KO; unc-85(cas1100) quadruple mutant animals (middle). Heatmap shows H3 depletion of H2B 3KO; unc-85(cas1100) quadruple mutant relative to WT (bottom). Dark blue boxes show the reference genes. (D) Immunofluorescence images with the anti-H4 antibody of WT, H2B 3KO, and H2B 3KO; unc-85(cas1100) quadruple mutant embryos. DAPI stained nuclei. Scale bar, 5 μm. (E) Quantification of histone H4 fluorescence intensity ratio relative to DAPI of WT, H2B 3KO, and H2B 3KO; unc-85(cas1100) quadruple mutant embryos. N = 43–101. Statistical significance compared with the control with a matching color code is based on Student’s t-test, n.s., not significant, ***p < 0.001. (TIF) [file pgen.1010223.s007.tif]

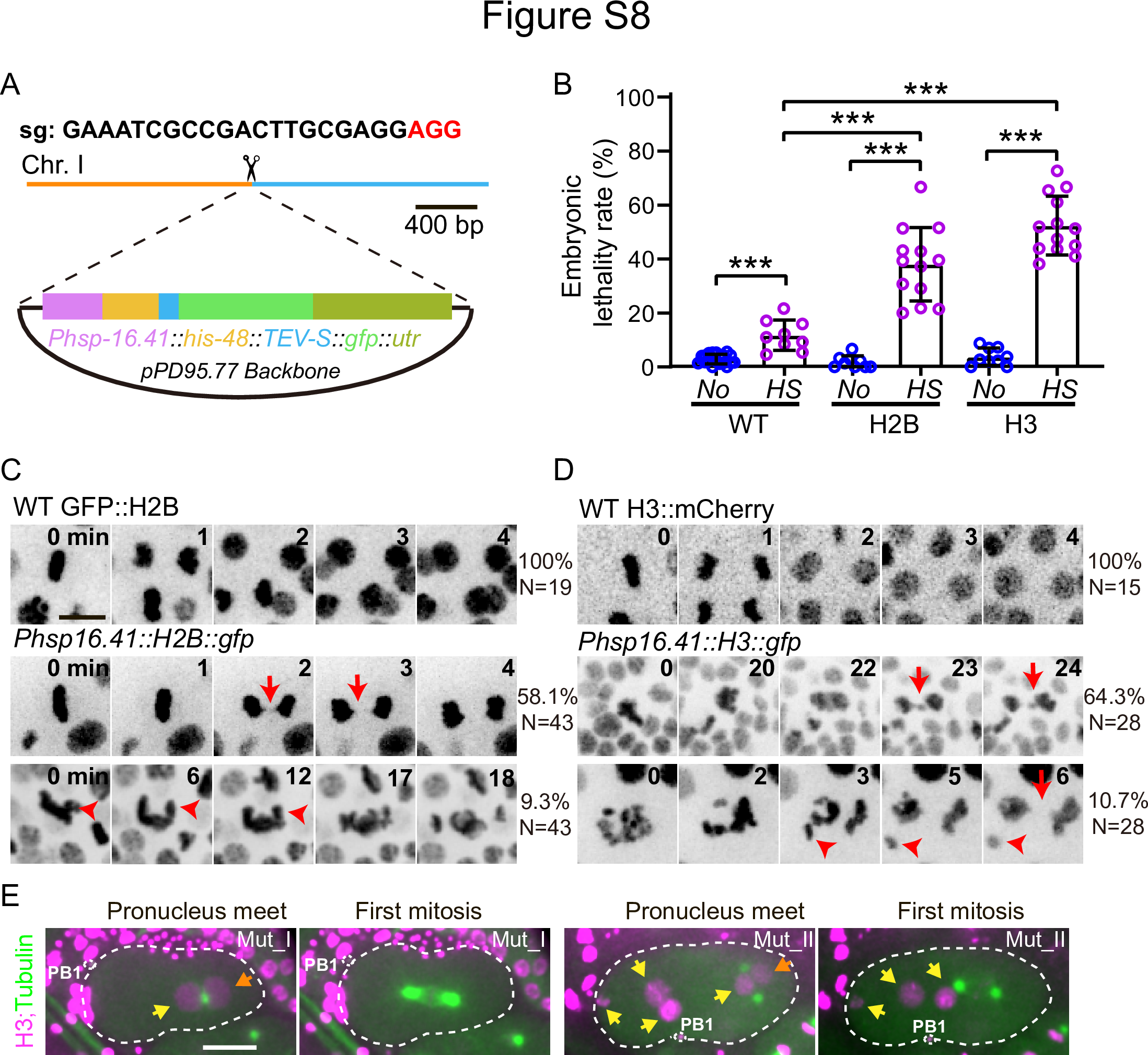

Supplement: S8 Fig — Related to Figs 4 and 5. (A) Schematic of hsp16.41::his-48::gfp single-copy insertion using the CRISPR-Cas9-based genome editing strategy. Scissor shows the Cas9 cleavage site, with the single-guide RNA (sgRNA) target sequence above and the PAM (Protospacer Adjacent Motif) sequence in red. A 5.6 kb homology arm was inserted into the plasmid pPD95.77 to make the homology recombination (HR) template. (B) Quantification of embryonic lethality rates in WT, H2B, and H3 overexpression alleles. Data are presented as mean ± SD (error bars). Statistical significance based on Student’s t-test, ***p < 0.001. N = 57–117 for H2B overexpression, N = 413–1242 for H3 overexpression. Data were from three biological replicates. (C) Fluorescence time-lapse images of GFP-tagged H2B during mitosis in WT and H2B overexpression embryos. Metaphase was defined as time zero. Scale bar, 5 μm. (D) Fluorescence time-lapse images of GFP-tagged H3 during mitosis in WT and H3 overexpression embryos. Metaphase was defined as time zero. Arrow indicates anaphase bridges, and arrowheads indicate the lagging chromatin. Percentages of meiosis patterns were indicated. Scale bar, 5 μm. (E) Fluorescence images of GFP-tagged tubulin with mCherry-tagged histone H3 and membrane (magenta) of one cell stage embryo in Mut I and Mut II at 25°C during pronucleus meet and first mitosis. Orange arrows show pronucleus from sperm, and yellow arrows show pronucleus from the oocyte. (TIF) [file pgen.1010223.s008.tif]
