# Supplementary material for: Inhibition of histone H3-H4 chaperone pathways rescues C. elegans sterility by H2B loss
Source: PLoS Genet. 2022 Jun 9;18(6):e1010223. doi: 10.1371/journal.pgen.1010223 (PMC9216614; doi:10.1371/journal.pgen.1010223)
Supplement: S1 Table — (DOCX) [file pgen.1010223.s015.docx]

S1 Table. *C. elegans* Strains in this study

| Strain name | Genotype | Method |
| --- | --- | --- |
| N2 | *Wild-type* | NA. |
| CB4856 | *Wild-type* | N.A. |
| GOU4367 | *his-48(cas976)IV in CB4856* | Microinjection |
| GOU3477 | *his-4(cas837)V;ujIs113[Ppie-1::H2B::mCherry,unc-119(+);Pnhr-2::HIS-24::mCherry, unc-119(+)]* | Genetic cross |
| GOU3478 | *his-8(cas838)V;ujIs113[Ppie-1::H2B::mCherry,unc-119(+);Pnhr-2::HIS-24::mCherry, unc-119(+)]* | Genetic cross |
| GOU3479 | *his-11(cas839)II;ujIs113[Ppie-1::H2B::mCherry,unc-119(+);Pnhr-2::HIS-24::mCherry, unc-119(+)]* | Genetic cross |
| GOU3480 | *his-15(cas840)II;ujIs113[Ppie-1::H2B::mCherry,unc-119(+);Pnhr-2::HIS-24::mCherry, unc-119(+)]* | Genetic cross |
| GOU3481 | *his-20(cas841)V;ujIs113[Ppie-1::H2B::mCherry,unc-119(+);Pnhr-2::HIS-24::mCherry, unc-119(+)]* | Genetic cross |
| GOU3482 | *his-22(cas842)V;ujIs113[Ppie-1::H2B::mCherry,unc-119(+);Pnhr-2::HIS-24::mCherry, unc-119(+)]* | Genetic cross |
| GOU3483 | *his-41(cas846)V;ujIs113[Ppie-1::H2B::mCherry,unc-119(+);Pnhr-2::HIS-24::mCherry, unc-119(+)]* | Genetic cross |
| GOU3484 | *his-44(cas847)II;ujIs113[Ppie-1::H2B::mCherry,unc-119(+);Pnhr-2::HIS-24::mCherry, unc-119(+)]* | Genetic cross |
| GOU3485 | *his-48(cas848)IV;ujIs113[Ppie-1::H2B::mCherry,unc-119(+);Pnhr-2::HIS-24::mCherry, unc-119(+)]* | Genetic cross |
| GOU3486 | *his-54(cas850)V;ujIs113[Ppie-1::H2B::mCherry,unc-119(+);Pnhr-2::HIS-24::mCherry, unc-119(+)]* | Genetic cross |
| GOU3487 | *his-58(cas851)IV;ujIs113[Ppie-1::H2B::mCherry,unc-119(+);Pnhr-2::HIS-24::mCherry, unc-119(+)]* | Genetic cross |
| GOU3488 | *his-62(cas852)IV;ujIs113[Ppie-1::H2B::mCherry,unc-119(+);Pnhr-2::HIS-24::mCherry, unc-119(+)]* | Genetic cross |
| GOU3489 | *his-29(cas843)IV;ujIs113[Ppie-1::H2B::mCherry,unc-119(+);Pnhr-2::HIS-24::mCherry, unc-119(+)]* | Genetic cross |
| GOU3490 | *UNC-85(cas990)II;ujIs113[Ppie-1::H2B::mCherry,unc-119(+);Pnhr-2::HIS-24::mCherry, unc-119(+)]* | Genetic cross |
| GOU4315 | *his-34(cas844)IV;ujIs113[Ppie-1::H2B::mCherry,unc-119(+);Pnhr-2::HIS-24::mCherry, unc-119(+)]* | Genetic cross |
| GOU4319 | *his-66(cas853)IV;ujIs113[Ppie-1::H2B::mCherry,unc-119(+);Pnhr-2::HIS-24::mCherry, unc-119(+)]* | Genetic cross |
| GOU3910 | *his-52(cas849)V;ujIs113[Ppie-1::H2B::mCherry,unc-119(+);Pnhr-2::HIS-24::mCherry, unc-119(+)]* | Genetic cross |
| GOU3911 | *his-39(cas845)V;ujIs113[Ppie-1::H2B::mCherry,unc-119(+);Pnhr-2::HIS-24::mCherry, unc-119(+)]* | Genetic cross |
| GOU3011 | *his-48(cas943)IV.* | Microinjection |
| GOU3012 | *his-58(cas946)IV.* | Microinjection |
| GOU3013 | *his-66(cas949)IV.* | Microinjection |
| GOU3014 | *his-66(cas950)IV.* | Microinjection |
| GOU3015 | *his-48(cas944)IV; his-58(cas946)IV.* | Microinjection |
| GOU3016 | *his-48(cas945)IV; his-58(cas947)IV.* | Microinjection |
| GOU3017 | *his-58(cas946)IV; his-66(cas951)IV.* | Microinjection |
| GOU3227 | *his-48(cas943)IV;his-58(cas948)IV;his-66(cas952)IV;nT1[qIs51](IV;V);his-72(thu95)III;tba-1(cas721)I* | Microinjection and genetic cross |
| GOU4312 | *his-72(thu95)III;tba-1(cas721)I* | Microinjection and genetic cross |
| GOU3948 | *unc-85(cas1100)II.* | EMS Screen |
| GOU3949 | *unc-85(cas1571)II.* | Microinjection |
| GOU3950 | *unc-85(cas1067)II.* | Microinjection |
| GOU3951 | *unc-85(cas1068)II.* | Microinjection |
| GOU4362 | *asfl-1(cas1222)I.* | Microinjection |
| GOU3908 | *his-48(cas943)IV;his-58(cas948)IV;his-66(cas952) IV;unc-85(cas1100) II;his-72(thu95);tba-1(cas721) I* | Genetic cross |
| GOU3841 | *his-48(cas943)IV;his-58(cas948)IV;his-66(cas952)IV;unc-85(cas1068) II;* | Genetic cross |
| GOU3842 | *his-48(cas943)IV;his-58(cas948)IV;his-66(cas952) IV;unc-85(cas1067) II;* | Genetic cross |
| GOU3886 | *his-48(cas943)IV;his-58(cas948)IV;his-66(cas952) IV;unc-85(cas1571)II;* | Genetic cross |
| GOU4363 | *his-48(cas943)IV;his-58(cas948)IV;his-66(cas952) IV; asfl-1(cas1222)I.* | Genetic cross |
| GOU3954 | *unc-85(cas990)II;ItIs44[pie-1p-mCherry::PH(PLC1delta1)+unc-119(+)];* | Microinjection and genetic cross |
| GOU3955 | *unc-85(cas1570)II;ItIs44[pie-1p-mCherry::PH(PLC1delta1)+unc-119(+)];* | Microinjection and genetic cross |
| GOU3926 | *his-74(cas1019)V;ItIs44[pie-1p-mCherry::PH(PLC1delta1)+unc-119(+)];* | Microinjection and genetic cross |
| GOU3928 | *his-48(cas943)IV;his-58(cas948)IV;his-66(cas952)IV;unc-85(cas1100)II;his-74(cas1019)V;ItIs44[pie-1p-mCherry::PH(PLC1delta1)+unc-119(+)];* | Microinjection and genetic cross |
| GOU4364 | *unc-85(cas1100)II;his-74(cas1019)V;ItIs44[pie-1p-mCherry::PH(PLC1delta1)+unc-119(+)];* | Microinjection and genetic cross |
| GOU4361 | *his-58(cas946)IV;his-66(cas951)IV; his-72(thu95)III;tba-1(cas721)I.* | Genetic cross |
| GOU4352 | *his-48(E74K)IV;his-58(cas1239)IV;his-66(cas1240)IV;his-72(thu95)III;tba-1(cas721)I.* | Microinjection and genetic cross |
| GOU4348 | *his-48(E74K)IV.* | Microinjection |
| GOU4357 | *his-48(E74K)IV;his-58(cas1239)IV;his-66(cas1240)IV;unc-85(cas1100)II;his-72(thu95)III;tba-1(cas721)I.* | Microinjection and genetic cross |
| GOU4365 | *casIs639(Phsp16.41-his-48::gfp) I.* | Microinjection |
| GOU4366 | *casIs640(Phsp16.41-his-44::gfp) I.* | Microinjection |
