# Supplementary material for: Inhibition of histone H3-H4 chaperone pathways rescues C. elegans sterility by H2B loss
Source: PLoS Genet. 2022 Jun 9;18(6):e1010223. doi: 10.1371/journal.pgen.1010223 (PMC9216614; doi:10.1371/journal.pgen.1010223)
Supplement: S2 Table — (DOCX) [file pgen.1010223.s016.docx]

**S2 Table. CRISPR-Cas9 Targets in this study**

| **Gene** | **CRISPR-Cas9 targets (PAM)** | **Application Description** |
| --- | --- | --- |
| *unc-85* | *Sg*1:  AGTGGCTCCGCCATCGATTCCGG | *unc-85* C-terminal *gfp* knock-in |
|  | *Sg*2: TCAGTGTTGATCTGAACGCCAGG |  |
|  | *Sg*3: CGTGGCAAATGAGTACACCGAGG | *unc-85* mutant |
|  | *Sg*4: GCGCTGCAAGTACAACGATCAGG |  |
|  | *Sg*5: TACTTGAACAATGTTGACACGGG | *unc-85* knock-out |
|  | *Sg*6: GTTTGTCGACAAATTCAAGTTGG |  |
| *asfl-1* | sg1: TCGCTGGATTATCGAGAATTTGG | *asfl-1* knock-out |
|  | sg2: AAGAATAATCAGATCTCGAATGG |  |
| *his-48* | sg: CCCTTGGCAGATGGCTTTGGTGG | *his-48* N-terminal *gfp* knock-in in CB4856 and onco-histone mutant |
| *his-74* | sg1: AAGAGTCACTATCATGCCAAAGG | *his-74* C-terminal *gfp* knock-in |
|  | sg2: AACTCGCCAGACGCATCCGTGG |  |
| *Chr.I single-copy sg* | sg: GAAATCGCCGACTTGCGAGGAGG | *Phsp16.41-his-48/his-45::gfp* single copy insert |
