# Supplementary material for: Inhibition of histone H3-H4 chaperone pathways rescues C. elegans sterility by H2B loss
Source: PLoS Genet. 2022 Jun 9;18(6):e1010223. doi: 10.1371/journal.pgen.1010223 (PMC9216614; doi:10.1371/journal.pgen.1010223)
Supplement: S3 Table — (DOCX) [file pgen.1010223.s017.docx]

**S3 Table. Plasmids and Primers in this study**

| Plasmid name | Forward primer | Reverse primer | notes |
| --- | --- | --- | --- |
| pDD162-P*eft-3::*  *Cas9+PU6::his-48 sg* | CCCTTGGCAGATGGCTTTGGGTTTTAGAGCTAGAAATAGCAAG | CCAAAGCCATCTGCCAAGGGCAAGACATCTCGCAATAGG | PCR from pDD162-P*eft-*  *3::Cas9+PU6::Empty sgRNA* |
| pPD95.77*-his-48* genomic template for knock-in | CCCTTGAGGGTACCGGTAGAAAAAGCAAACGCTGGTCCACAGCG | GGGATGTTGAAGAGTAATTGGACCCAAGAACATGAAGCGGAAG | *his-48* genomic fragment was amplified from N2 and cloned into pPD95.77 |
| pPD95.77-*his-48::gfp* template for Knock-in | GATTCAAGTTCATTCTCATCATGAGTAAAGGAGAA | GCAGATGGCTTTGGTGGCATACCGCTACCACTTCCAGCTTTGTATAGTTCATCCATG | *gfp* fragment was amplified and cloned into pPD95.77  *-his-48* template |
| pPD95.77-*his-48(E74K)* template | CGCTTCGAAAGCTTCCCGTCTTGCT | GGGAAGCTTTCGAAGCGATGCGTTCG | *his-48(E74K)* was introduced into pPD95.77*-his-48*  genomic template |
| pPD95.77-*his-48(E74K)* template | TCCCCGGGGAACTTGCCAAGCACGCCGT | CCCCGGGGAGAATCAAACGGACAGCGGT | Synonymous mutations wer introduced into pPD95.77*-his-48(E74K)* genomic template to generate *Sal I* restriction enzyme cutting site |
| pPD95.77-*his-48(E74K)* template | CCTAAACCTAGCGCAAAAGGAGCCAAGAAGGCCGCCAAGA | CCTTTTGCGCTAGGTTTAGGTGGCATGATGAGAATGAACTTGAATC | Synonymous mutations were introduced into pPD95.77*-his-48(E74K)* genomic template to sgRNA target site |
| *his-48(E74K) genotyping* primer | TCTAGAATTTCCTCATACGTTT | TTCCCGAACCATATGAATTCAC | Identifying *his-48(E74K) mutant* |
| pDD162-P*eft-3::*  *Cas9+PU6::unc-85 sg1* | AGTGGCTCCGCCATCGATTCGTTTTAGAGCTAGAAATAGC | GAATCGATGGCGGAGCCACTCAAGACATCTCGCAATAGGA | PCR from pDD162-P*eft-*  *3::Cas9+PU6::Empty sgRNA* |
| pDD162-P*eft-3::*  *Cas9+PU6::unc-85 sg2* | TCAGTGTTGATCTGAACGCCGTTTTAGAGCTAGAAATAGC | GGCGTTCAGATCAACACTGACAAGACATCTCGCAATAGGA | PCR from pDD162-P*eft-*  *3::Cas9+PU6::Empty sgRNA* |
| pPD95.77*-unc-85*  genomic template for knock-in | TTGAGGGTACCGGTAGAAAAATGCCAATACTGTTGTGAATTG | TAGGGATGTTGAAGAGTAATTGGACTCTGTTGGACAAATTTGGAAGAAC | *unc-85* genomic fragment was amplified from N2 and cloned into pPD95.77 |
| pPD95.77-*unc-85::gfp* template for Knock-in | CAAACAACGAAATGGTCCAGGCTGGAAGTGGTAGCGGTATGAGTAAAGGAGAAGAACTTTTC | TAAATTAACTGGAAGTGAAACTATTTGTATAGTTCATCCATGCC | *gfp* fragment was amplified and cloned into pPD95.77  *-unc-85* template |
| *unc-85::gfp* Knock-in genotyping inner primer | TGGCCAATGCACTTGACGGG | AGTAGAACTCGCTAGACATT | Identifying *unc-85::gfp* Knock-in |
| *unc-85::gfp* Knock-in genotyping outer primer | GAAGCGTTCAACTAGCAG | AAGCTGCTGCTCGAGAGGCTGC | Identifying *unc-85::gfp* Knock-in |
| pDD162-P*eft-3::Cas9+PU6::unc-85 sg3* | CGTGGCAAATGAGTACACCGGTTTTAGAGCTAGAAATAGC | CGGTGTACTCATTTGCCACGCAAGACATCTCGCAATAGGA | PCR from pDD162-P*eft-*  *3::Cas9+PU6::Empty sgRNA* |
| pDD162-P*eft-3::Cas9+PU6::unc-85 sg4* | GATCGTTGTACTTGCAGCGCGTTTTAGAGCTAGAAATAGC | GCGCTGCAAGTACAACGATCCAAGACATCTCGCAATAGGA | PCR from pDD162-P*eft-*  *3::Cas9+PU6::Empty sgRNA* |
| pPD95.77*-unc-85*  genomic template for mutant | ATGGTTTGTCGCTAACGAATATACGGAAGAAGAGCTCAAAGAGAA | CTCTTCTTCCGTATATTCGTTAGCGACAAACCAT | Synonymous mutations were introduced into pPD95.77*-unc-85* genomic template to sgRNA target site |
| pPD95.77*-unc-85*  genomic template for *unc-85(G111K)* mutant | ATGGTTTGTCGCTAACGAATATACGGAAGAAGAGCTCAAAGAGAA | GCTCTTCTTCCGTATATTCGTTAGCGACAAACCATTTCATATTAATA | Synonymous mutations was introduced into pPD95.77*-unc-85* genomic template to generate mutant and *PshB I* restriction enzyme cutting site |
| pPD95.77*-unc-85*  genomic template for *unc-85(G111E)* mutant | ATGGTTTGTCGCTAACGAATATACGGAAGAAGAGCTCAAAGAGAA | CTCTTCTTCCGTATATTCGTTAGCGACAAACCATTCCATATTAATAA | Synonymous mutations was introduced into pPD95.77*-unc-85* genomic template to generate mutant and *PshB I* restriction enzyme cutting site |
| pDD162-P*eft-3::Cas9+PU6::unc-85 sg5* | TACTTGAACAATGTTGACACGTTTTAGAGCTAGAAATAGCA | GTGTCAACATTGTTCAAGTACAAGACATCTCGCAATAGGAG | PCR from pDD162-P*eft-*  *3::Cas9+PU6::Empty sgRNA* |
| pDD162-P*eft-3::Cas9+PU6::unc-85 sg6* | GTTTGTCGACAAATTCAAGTGTTTTAGAGCTAGAAATAGCA | ACTTGAATTTGTCGACAAACCAAGACATCTCGCAATAGGAG | PCR from pDD162-P*eft-*  *3::Cas9+PU6::Empty sgRNA* |
| pDD162-P*eft-3::Cas9+PU6::asfl-1 sg1* | TCGCTGGATTATCGAGAATTGTTTTAGAGCTAGAAATAGCA | AATTCTCGATAATCCAGCGACAAGACATCTCGCAATAGGAG | PCR from pDD162-P*eft-*  *3::Cas9+PU6::Empty sgRNA* |
| pDD162-P*eft-3::Cas9+PU6::asfl-1 sg2* | AAGAATAATCAGATCTCGAAGTTTTAGAGCTAGAAATAGCA | TTCGAGATCTGATTATTCTTCAAGACATCTCGCAATAGGAG | PCR from pDD162-P*eft-*  *3::Cas9+PU6::Empty sgRNA* |
| pDD162-P*eft-3::Cas9+PU6::his-74 sg1* | AAGAGTCACTATCATGCCAAGTTTTAGAGCTAGAAATAGCA | TTGGCATGATAGTGACTCTTCAAGACATCTCGCAATAGGAG | PCR from pDD162-P*eft-*  *3::Cas9+PU6::Empty sgRNA* |
| pDD162-P*eft-3::Cas9+PU6::his-74 sg2* | AACTCGCCAGACGCATCCGGTTTTAGAGCTAGAAATAGCA | CGGATGCGTCTGGCGAGTTCAAGACATCTCGCAATAGGAG | PCR from pDD162-P*eft-*  *3::Cas9+PU6::Empty sgRNA* |
| pPD95.77*-his-74* genomic template for knock-in | CCCTTGAGGGTACCGGTAGAAAAACGATCCAAGCTATAAATCTC | GGGATGTTGAAGAGTAATTGGACTCGGCACTTGTCACCAAATCG | *his-74* genomic fragment was amplified from N2 and cloned into pPD95.77 |
| pPD95.77-*his-74::gfp* template for Knock-in | CCGGTGGCGGCCGCTCTAGAGAGAATCTTTATTTTCAGGGC | CGGGCAGTAATAAAACAGGGATCTATTTGTATAGTTCATCCATGCCATG | *TEV-S-gfp* fragment was amplified and cloned into pPD95.77*-his-74* template |
| *his-74::gfp* Knock-in genotyping inner primer | GCAAGACTTCAAAACCGATC | ACTCTTCTAACGTAAATCCACTC | Identifying *his-74::gfp* Knock-in |
| *his-74::gfp* Knock-in genotyping outer primer | GAAGCGTTCAACTAGCAG | GCAAAGCAAAGCGTCTTCTGGTC | Identifying *his-74::gfp* Knock-in |
| pDD162-Peft-3::Cas9+PU6:: Chr.I single-copy sg1 | GAAATCGCCGACTTGCGAGGGTTTTAGAGCTAGAAATAGCA | CCTCGCAAGTCGGCGATTTCCAAGACATCTCGCAATAGGAG | *PCR from pDD162-Peft-*  *3::Cas9+PU6::Empty sgRNA* |
| pPD95.77- Chr.I single-copy genomic template | CCCTTGAGGGTACCGGTAGAAAAATTAGGGTGCAAGTTTTTTAT | GGGATGTTGAAGAGTAATTGGACCAAGTGGGGATCAGGAAGAAG | Chr.I single-copy *genomic fragment was amplified from N2 and cloned into pPD95.77* |
| pPD95.77- Chr.I single-copy arm-*TEV-S-gfp* genomic template | CTTCAAAGAAATCGCCGACTTTGCCCGGGGGATCGGTGGAGCT | GGGTAGTTTTCAGCCTAATGGCTTATTTGTATAGTTCATCCATGCC | *TEV-S-gfp* fragment was amplified and cloned into pPD95.77- Chr.I single-copy genomic template |
| pPD95.77- Chr.I single-copy arm-*his-48*-*TEV-S-gfp* genomic template | CTTCAAAGAAATCGCCGACTTATGCCACCAAAGCCATCTGC | AGCTCCACCGATCCCCCGGGCACTTGCTGGAAGTGTACTTGG | *his-74* *genomic fragment was amplified from N2 and cloned into pPD95.77- Chr.I single-copy arm-TEV-S-gfp genomic template* |
| pPD95.77- Chr.I single-copy arm-*hsp16.41-his-48*-*TEV-S-gfp* genomic template | CTTCAAAGAAATCGCCGACTTGATCACCAAAAACGGAACGTTG | GCAGATGGCTTTGGTGGCATTTTCGAAGTTTTTTAGATGCAC | *Phsp16.41 genomic fragment was amplified from N2 and cloned into* pPD95.77- Chr.I single-copy arm-*his-48*-*TEV-S-gfp* genomic template |
| pPD95.77- Chr.I single-copy arm-*hsp16.41-his-45*-*TEV-S-gfp* genomic template | GTGCATCTAAAAAACTTCGAAAATGGCTCGTACCAAGCAAAC | AGCTCCACCGATCCCCCGGGCAAGCGCGCTCTCCTCGGATGCG | *his-45 genomic fragment was amplified from N2 and cloned into* pPD95.77- Chr.I single-copy arm-*his-48*-*TEV-S-gfp* genomic template |
| pCMV*-his-48-Scarlet* | GATATCGCGGCCGCTCTAGAATGCCACCAAAGCCATCTGCCA | ACCGCTACCACTTCCAGCCTTGCTGGAAGTGTACTTGG | *his-48* genomic fragment was amplified from N2 and cloned into pCMV-Scarlet |
| pCMV*-his-48(E74K)-Scarlet* | ATCGCTTCGAAAGCTTCCCGTCTTGCTCATTA | GACGGGAAGCTTtCGAAGCGATGCGTTCGAATA | *his-48(E74K)* was introduced into pCMV-*his-48*-Scarlet |
