# Supplementary material for: Inhibition of histone H3-H4 chaperone pathways rescues C. elegans sterility by H2B loss
Source: PLoS Genet. 2022 Jun 9;18(6):e1010223. doi: 10.1371/journal.pgen.1010223 (PMC9216614; doi:10.1371/journal.pgen.1010223)
Supplement: Attachment — Submitted filename: Response to reviewers_20220410.doc [file pgen.1010223.s018.doc]

Apr 10, 2022

*PLOS Genetics*

Tracking #: PGENETICS-D-22-00207

Authors: Zhao et al.

Dear Prof. William G. Kelly,

Thank you for your letter of Mar 22, 2022. I now submit our revised manuscript entitled "Inhibition of Histone H3-H4 Chaperone Pathways Rescues *C. elegans* Sterility by H2B Loss " for publication in *PLOS Genetics.*

We are glad that reviewers and the journal are interested in the topic and our work. We appreciate that you and the reviewers suggested that we remove the human cell studies from the manuscript. We followed the advice and changed the title, specifying that the findings were from *C. elegans* in the loss of H2B. As detailed in response to reviewers, we have completed the suggested experiments, and our results support our conclusion. We have also carefully addressed each point through data analysis and textual clarifications.

We appreciate the comments from you and the reviewers. We thank you for your time and attention and for handling our manuscript.

Sincerely,

Guangshuo

Guangshuo Ou, Ph.D.

Professor

Tsinghua-Peking Center for Life Sciences

School of Life Sciences

Tsinghua University, Beijing 100084, China

E-mail: [guangshuoou@tsinghua.edu.cn](mailto:guangshuoou@tsinghua.edu.cn)

RESPONSE TO THE REVIEWERS

Reviewer #1: In this manuscript, Zhao et al use C. elegans to investigate the consequences of H2B loss in the germline and early embryos. First and perhaps not surprisingly, they find that H2B triple KO of 3/4 paralogues that are expressed in these cells results in sterility. Interestingly, they then perform suppressor screens and identify that loss of H3-H4 chaperones can restore fecundity. A particularly intriguing finding is that knockdown HIRA or ATRX/DAXX both restore fecundity even though they are responsible for H3.3 deposition in distinct types of chromatin, and that CAF-1 knockdown, which is responsible for replication-coupled deposition primarily of H3.1, also restores knockdown. Thus, impairing any H3 deposition pathway rescues this phenotype. They identify defects in meiosis in the H2B 3KO worms, and that H3 chaperone knockdown restores proper chromosome segregation during meiosis. Finally, in an attempt to link their H2B KO findings to human disease, they examine H2BE76K (74K in worms) effects on fertility and find that only in the context of the H2B 2KO does the H2B-E74K have an effect, which is more modest than the 3KO. They also express H2B-E74K in HEK293 cells and find a subtle mitotic delay phenotype. The C. elegans work and cleanliness of the system, as well as simplicity of these experiments, are convincing in demonstrating both that loss of H2B causes sterility and that H3/H3 chaperone knockdown rescues this phenotype. The work in 293 cells is more complicated and the relevance to their C. elegans findings is less clear. This is in part because in the 293 context the mutant histone is overexpressed and likely a small fraction of the total H2B, whereas in the worm experiments H2B-E74K comprises a much larger fraction of the total H2B pool in the 2KO background. Further, the 293 phenotype is very mild and it appears that the variance in MI is more impacted than the number of cells in mitosis. Finally, 293 cells have a high rate of MI at baseline. The link between H2B KO and H2B-E74K is also somewhat more complicated because H2B-E76K destabilizes nucleosomes, which is different than not making H2B in the first place. However, the screen results are fascinating and I think this work stands alone without the human experiments, but if those are to be included they should be strengthened.

We thank the reviewer for the positive comments on our study. We appreciate that the reviewer thinks that the *C. elegans* results stand alone. As editor and reviewer #3 suggested, we have removed the human cell experiments.

Minor Points:

1. If intending to include the human work, an orthogonal assay (e.g. flow cytometry w/DAPI) to examine cell cycle progression in H2B-E76K expressing 293T cells to show whether cells are accumulating in M. Can be coupled with H3S10 or S28 staining as is done in commercially available assays. The DAPI result is important because the Asf-1 rescue could be explained by less H3S10 deposition in chromatin as a result of less H2B deposition in chromatin.

We have removed the human cell experiments.

2. Western blotting for H2B-E76K expression in 293 should be done (I presume the constructs are tagged) and anti-H2B WB should be used to assess expression of the mutant (or WT control) vs total H2B pool.

We have removed the human cell experiments.

3. Not essential, but can the authors stain for CENP-A and count foci in 293 cells to determine whether deposition is impacted? This might explain the mitotic phenotype although HJURP is the CENP-A chaperone, not Asf-1.

We have removed the human cell experiments.

4. The word "chaperone" is misspelled several times, particularly in the paragraphs the data in figure 5.

We apologize for the misspelling. We have corrected them and thank the reviewer for pointing this out.

Reviewer #2: The manuscript titled “Inhibition of Histone H3-H4 Chaperone Pathways Rescues Dysfunctional Oncohistone H2B” by Guangshuo Ou and co-authors provides an important contribution to our understanding of chromatin dynamics in animals.

The key conclusions of the authors are well supported by the presented data. Specifically, they establish in an unbiased manner that sterility of C. elegans lacking several H2B genes is not due to the histone/nucleosome depletion per se, but is due to the disruption of stoichiometry between H2A/H2B dimers and H3-H4 tetramers. Furthermore, the authors model oncogenic H2B (E76K) mutation that destabilizes nucleosome core in C. elegans and show the potential of H3-H4 chaperone loss in rescuing the sterility phenotype. In parallel, it is shown that H2B (E76K)-driven mitotic arrest in human cell culture can be rescued by H3-H4 chaperone downregulation. The latter result suggest a possibility of using histone chaperone inhibitors for treatment of some cancers caused by oncohistones.

Scientifically, I support publication of the manuscript. I have concerns regarding manuscript language that often affects meaning and some other suggestions/corrections.

We appreciate that the reviewer is positive about our work.

I was not able to evaluate GEO submission without the token number.

We have updated the GEO submission and released the data from Apr 03, 2022.

Specific comments:

1. Title. The title only reflects the oncohistone part of the story, a more general one referring to the importance of H2A/H2B and H3-H4 balance may work better.

We thank the reviewer for the suggestion on the title. Indeed, we would like to use a more general one referring to the importance of H2A-H2B and H3-H4 balance. Through our discussion with biochemists in the field, they argued that we did not directly quantify the balance. Therefore, our revised title, “Inhibition of Histone H3-H4 Chaperone Pathways Rescues *C. elegans* Sterility by H2B Loss,” accurately describes our major conclusion, mainly from genetics. Nevertheless, in the revised Summary, we have indicated “…the overall stoichiometry of histones within nucleosomes is more important than the actual abundances of histone dimers or tetramers….”

2. Summary has to be edited for word omission. Also, “fertility” works better than “animal defects”.

We have edited the Summary as the following and replaced animal defects with fertility.

“Dysfunctional histones cause developmental defects and drive tumorigenesis. Using *Caenorhabditis elegans* to study histone H2B loss, we found that inhibiting histone H3-H4 chaperones restored fertility in H2B triple knockouts by decreasing chromatin H3-H4 levels. The blockade of H3-H4 chaperones recovered cell division in *C. elegans* carrying the oncohistone H2B^E74K^ mutation that distorts the H2B-H4 interface and induces nucleosome instability. We suggest that the overall stoichiometry of histones within nucleosomes is more important than the actual abundances of histone dimers or tetramers and that inhibiting H3-H4 chaperones may provide an effective therapeutic strategy for treating cancers resulting from loss-of-function H2B oncohistone.”

3. Introduction. The contrast between the statements in sentence 3 “The best-….” and sentence 4 “In contrast, ….” is not clear. Did the authors mean to contrast oncomutations that disrupt proper histone tail PTMs with those affecting nucleosome structure?

Yes. That’s what we want to mean. We have clarified the writing as the following: “……In contrast to the well-known PTM-associated mutations, the most common oncohistone mutations localize in the globular histone fold domain……” on page 5, lines 71-72 in the marked-up copy.

4. “facilitated us” has to be changed

We have made the change as the following: “enabled us” on page 6, line 102, and page 14, line 310 in the marked-up copy.

5. Page 6: Here and throughout the manuscript “loss of UNC-85 restored sterility in H2B 3KO animals”; change to “restored fertility” or “rescued the sterility”, also “rescue meiosis defects”.

We have changed the writing to “restored fertility.” We thank the reviewer for pointing this out to us.

6. Page 8: change “reduced histone H3 proteins from chromatin”

We have changed the following: “……whether the loss of H3-H4 chaperone reduced histone H3 proteins in the nucleus…….” on page 10, line 215 in the marked-up copy.

7. Methods. Molecular dynamic simulation. Can this section be described in a more comprehendible manner for readers like me? “protein constructs were placed in a cubic box of 100 nM NaCl in simple point charge water” reads like “wet lab” experiment description. Change to “protein models”? “simulation of a cubic box with 100 nM NaCl”?

We have described in a more comprehendible manner on page 18 and 19 in the marked-up copy:

“Wild-type yeast Asf1-H3-H4 complex (PDB code: 2HUE) and free Asf1 (PDB code: 1ROC) structures were downloaded from PDB, and G110R mutation structures were created by USCF Chimera (RRID:SCR_004097) with energy minimization. Molecular dynamics simulations were performed using GROMACS (RRID:SCR_014565) version 4.6.7 using the OPLS-AA force field parameter set. After dispersion correction, each system was solvated in 150 mM NaCl solvent with SPC/E water models in a cubic box. Subsequently, neutralizing counter ions were added, and the steepest descent energy minimization was performed, followed by a two-step equilibration consisting of 100 ps of isochoric-isothermal (NVT) equilibration and 100 ps of isothermal-isobaric (NPT) equilibration. All position restraints were removed, and simulations were performed for 5 ns. RMSF and RMSD analysis calculate the standard deviation (root mean square fluctuation/deviation) off the atomic positions of the specified amino acids compared to their starting position within the energy minimized and equilibrated structure. Simulations were performed in triplicate; the values shown in the figures are averages with a standard deviation of the mean highlighted as error bars. Structural analysis and images were performed and created using PyMol (RRID:SCR_000305). The electrostatic distribution surfaces were generated using the APBS tool (RRID:SCR_008387).”

8. Methods. Chromatin Immunoprecipitation. Change “grind worms into frozen powder….” sentence.

We have changed the following: “……. Then, we ground the worm "popcorn" into frozen powder with a mortar and crosslinked with 1.1% formaldehyde for 15 min at room temperature…....” on page 20, line 468 in the marked-up copy.

9. Methods. Chromatin Immunoprecipitation. “We reversed the crosslink with Proteinase K”. Formaldehyde crosslinking is reversed by heat, i.e. 30 min at 65C.

We have made this correction on page 21, line 490 in the marked-up copy.

10. References. #18: First names of the authors are listed instead of their last names. Douglas Meeks-Wagner and Leland H.Hartwell.

We have corrected the format of the References on page 30, line 751 in the marked-up copy.

11. Figure 1 legend. (B) include “endogenously GFP-tagged”, (D) “proteins” plural, (E) only a portion of protein sequences is aligned; what is the significance of this domain?

We have made the change as reviewer’s suggestions in Figure 1 legend on page 32, line 809, and line 814 in the marked-up copy;

We have explained why we provided a small portion of the aligned protein sequences as below in the revised text on page 32, lines 818-821 in the marked-up copy:

“…The sequence alignment shows the protein sequence of UNC-85 histone chaperone domain flanking (± 20 aa) around the mutation isolated from the EMS screen, and the histone chaperone domain is highly acidic, which favors its interaction with histone….”

12. Figure 2 (F). Provide the number of oocytes scored, and number of animals used.

We have provided the numbers in the revised Figure 2 (F) legend: N (N=24-61 animals, oocytes are from different animals), and percentages of meiosis patterns were indicated on the right on page 33, lines 845-846 in the marked-up copy.

13. Include “Statistical significance compared with the control with a matching

color code” explanation in legends to Figures 3, 5, etc.

We have included this statement.

14. Figure 4D, I do not understand the legend. The Y axis is labeled “genes”, is this correct. The “gene distance bp” on the x-axis can correspond to the 3Kb flanks of genes, not to the gene model.

We have clarified the legend as the following: The labeled “genes” is the Y-axis of Figure 4C; We have changed the Y-axis “genes” to the left of Figure 4C to avoid confusion.

14. Figure 4E. I suggest replacing “H3 enrichment” with “H3 depletion” to better convey the point, same for relevant supplemental figures.

We have performed the replacement in these places on page 35, line 871, and page 25, line 616 in the marked-up copy.

15. Figure S3. Indicate that DNA is shown in magenta, since on figure S2 histone H2B is shown in this color.

We have changed the color of Figure S3 as the reviewer suggested.

16. Figure S5. I do not think panels G, I, J are necessary.

We have removed these panels.

17. Figure S6. Change to “rescue sterility”

We have made the change and thank the reviewer for pointing out all these issues.

Reviewer #3: In this manuscript, the authors report that by blocking H3-H4 chaperones like UNC-85, ASFL-1 and others, they were able to restore defects in H2B 3KO C. elegans, including cell division and fertility defects. Then they extended to the claim that the inhibition of H3-H4 chaperones largely restore H2BE76K-induced defects in both C. elegans and HEK293 cells. However, the developmental defects are more likely caused by insufficient H2B supply rather that E74K mutant in C. elegans.

The human cell experiments appear to be not as solid and I suggest to remove them or to provide substantial amounts of supporting evidence.

We appreciate that the reviewer is also favorable to our work. We have removed the human cell experiments.

Major points:

1. HIRA and DAXX complexes are responsible for depositing H3.3 into transcriptional active regions and telomeric/centromeric regions, respectively. On the other hand, CAF-1 is required for replication-coupled nucleosome assembly. Why knockdown of all these chaperones with distinct functions sucessfully rescued H2B 3KO defects? Any explanations?

We thank the reviewer for asking this question and carefully discussed this issue in the revised text on page 10, lines 196-202 in the discussion in the marked-up copy.

“…Our data revealed that inhibition of the H3-H4 chaperones with distinct functions rescued the sterility of H2B 3KO embryos. Like many other species, the *C. elegans* early embryos do not undergo active gene transcription until the eight-cell stage, excluding the possibility that these chaperones play distinct regulatory roles in gene expression. Instead, we suspect that the blockade of any chaperone will reduce the H3H4 amount from chromatin, restoring the overall stoichiometry of histones within nucleosomes close to the normal level….”

2. In humans, H2B is coded by dozens of genes, and histones are highly abundant in human cells. It is hard to imagine that severe mitotic defects (56%, Fig. 5E) can be caused by the overexpressed H2B E74K, unless the mutation has a very strong dominant effect. If that is the case, cas1172(E74K) alone should lead to brood size defects. What is the phenotype when E74K occurs on only one histone H2 protein (like his-48E74K, not at the background of his-58/his-66 dKO)?

We have removed the human cell experiments. Probably overexpression exaggerated the H2B E74K effects in human cells; however, in *C. elegans,* *cas1172(E74K)* was generated by genome editing, the low endogenous expression of which may not be able to create apparent defects. Indeed, we did not observe that E74K affected animal development in WT background, as shown in Fig 5C (Mut III), likely because other normal H2B proteins compensate for a small portion of abnormal H2B^E74K^. We have discussed this issue in the revised text on page 12, lines 272-279 in the marked-up copy.

“…We did not notice that the single HIS-48^E74K^ mutation causes apparent sterility, likely due to the functional redundancy of histone genes. We speculate that the other three wild-type H2B genes generate normal H2B proteins that sustain embryo development in the *his-48^E74K^* single mutant. However, if we deleted another two H2B genes, i.e., *his-58 ^KO^* and *his-66 ^KO^*, the only left WT H2B gene may not produce a sufficient amount of normal H2B protein to maintain the proper chromatin structure that supports early development in *his-48^E74K^*, *his-58^KO^* and *his-66^KO^* triple mutant embryos…. ”

3. Fig. S8A-8D, the authors used heat-shock system to express histones in the embryos. However, the level of overexpression is not determined. In addtion, a negative control that only overexpresses GFP is missing.

We thank the reviewer for pointing out the caveat of this experiment. Indeed, overexpression of a particular protein in the *C. elegans* early embryos with a constant level is not a trivial experiment because of the well-known germline silence of foreign genes. Nevertheless, we achieved the overexpression by knocking the heat-shock system into the *C. elegans* genome. The expression level should be constant because the single copy of the transgene was integrated into the genome. In addition, overexpressed GFP protein alone has not been known to be toxic for animal development. The *C. elegans* field routinely uses overexpressed GFP to mark cells or label proteins for decades without noticing significant side effects. However, the heat shock of the developing embryos will cause 10~20% lethality in our hands and many other labs. Because failures of cell division by overexpressed histone proteins have been documented in several different species, this set of *C. elegans* experiments confirmed the primary conclusion from early studies. Therefore, we have now cited the early results and acknowledged the caveat from heat shock in the revised discussion on page 11, lines 241-248 in the marked-up copy:

“ Using the heat shock overexpression system, we observed that H2B or H3 overexpressed *C. elegans* embryos underwent abnormal chromatin segregation and defective cell division, which is consistent with the early results that overexpressed histone genes disrupted cell division in yeast and human cells [17-19]. Although we observed markedly higher penetrance of cell division failure in embryos overpressing histone than the wild-type embryos treated by heat shock, we cannot exclude the effects of heat shock on embryonic development because heat shock of WT embryos caused 10~20% embryonic lethality [20, 21].….”

Minor points:

1. Third line of figure legend in Fig. S7, it is suggested to use quadruple KO or quadruple mutant instead of "double mutant", which may cause confusion about the genotype of the animal.

We have used quadruple mutant to replace double mutant on page 25, line 609 in the marked-up copy. We thank the reviewer for pointing this out to us.

2. KD efficiency of Asf1a and Asf1b in HEK293T cells should be verified.

We have removed the human cell experiments.

3. In Fig 5C and 5D, a WT control should be added.

We have provided a WT control in Fig 5C and 5D.
